# Supplementary material for: Molecular Responses to Thermal and Osmotic Stress in Arctic Intertidal Mussels (Mytilus edulis): The Limits of Resilience
Source: Genes (Basel). 2022 Jan 15;13(1):155. doi: 10.3390/genes13010155 (PMC8774603; doi:10.3390/genes13010155)

Supplementary material for:

Molecular responses to thermal and osmotic stress in Arctic intertidal mussels (*Mytilus edulis*): the limits of resilience

Nicholas J. Barrett, Jakob Thyrring, Elizabeth M. Harper, Mikael K. Sejr, Jesper G. Sørensen, Lloyd S. Peck and Melody S. Clark

Supplementary Table S1. Accession numbers for aquaporin genes used in the phylogenetic analysis (see Figure 4).

Accession number	Animal	Aquaporin gene
NP_776362	Cattle	Aquaporin-0 [Bos taurus]
P47865.3	Cattle	Aquaporin-1 [Bos Taurus]
NP_001094669.1	Cattle	Aquaporin-2 [Bos Taurus]
Q08DE6.1	Cattle	Aquaporin-3 [Bos Taurus]
AAI18416.1	Cattle	Aquaporin 4 [Bos Taurus]
NP_001178089.1	Cattle	Aquaporin-5 [Bos Taurus]
XP_002687325.1	Cattle	Aquaporin-6 [Bos Taurus]
NP_001069846.1	Cattle	Aquaporin-7 [Bos Taurus]
NP_001193536.1	Cattle	Aquaporin-8 [Bos Taurus]
XP_015328630.1	Cattle	Aquaporin-9 Isoform X2 [Bos Taurus]
XP_024845589.1	Cattle	Aquaporin-10 [Bos Taurus]
NP_001103539.1	Cattle	Aquaporin-11 [Bos Taurus]
XP_005205120.1	Cattle	Aquaporin-12 [Bos Taurus]
AAC52416	House mouse	Aquaporin-0_[Mus musculus]
EDK98728.1	House mouse	Aquaporin 1 [Mus Musculus]
AAD21017.1	House mouse	Aquaporin 2 [Mus Musculus]
EDL05416.1	House mouse	Aquaporin 3 [Mus Musculus]
EDL01600.1	House mouse	Aquaporin 4, Isoform Cra_A [Mus Musculus]
AAD32491.1	House mouse	Aquaporin 5 [Mus Musculus]
ABH01263.1	House mouse	Aquaporin 6 [Mus Musculus]
EDL05417.1	House mouse	Aquaporin 7, Isoform Cra_A [Mus Musculus]
EDL17310.1	House mouse	Aquaporin 8, Isoform Cra_A, Partial [Mus Musculus]
EDL26217.1	House mouse	Aquaporin 9, Isoform Cra_A [Mus Musculus]
EDL16320.1	House mouse	Aquaporin 11, Isoform Cra_A [Mus Musculus]
EDL39974.1	House mouse	Aquaporin 12, Isoform Cra_A [Mus Musculus]
P30301.1	Human	Aquaporin_0_Homo sapiens
NP_932766.1	Human	Aquaporin-1 Isoform 1 [Homo Sapiens]
NP_000477.1	Human	Aquaporin-2 [Homo Sapiens]
AAP35863.1	Human	Aquaporin 3 [Homo Sapiens]
NP_001641.1	Human	Aquaporin-4 Isoform M1 [Homo Sapiens]
EAW58110.1	Human	Aquaporin 5 [Homo Sapiens]
Q13520.2	Human	Aquaporin-6 [Homo Sapiens]
EAW58505.1	Human	Aquaporin 7, Isoform Cra_A [Homo Sapiens]
NP_001160.2	Human	Aquaporin-8 [Homo Sapiens]
BAA24864.1	Human	Aquaporin 9 [Homo Sapiens]
AAH74896.1	Human	Aquaporin 10 [Homo Sapiens]
BAC45004.1	Human	Aquaporin 11 [Homo Sapiens]
Q8IXF9.1	Human	Aquaporin-12A [Homo Sapiens]
XP_021364227.1	Yesso scallop	Aquaporin-1-Like Isoform X1 [Mizuhopecten Yessoensis]
XP_021373368.1	Yesso scallop	Aquaporin-8-Like [Mizuhopecten Yessoensis]
XP_021359304.1	Yesso scallop	Aquaporin-9-Like Isoform X1 [Mizuhopecten Yessoensis]
XP_021370233.1	Yesso scallop	Aquaporin-11 [Mizuhopecten Yessoensis]
FJ666326	Zebrafish	Aquaporin-0_ [Danio rerio]
AAV34608.1	Zebrafish	Aquaporin 1 [Danio Rerio]
AAH44188.1	Zebrafish	Aquaporin 3 [Danio Rerio]
NP_001003749.1	Zebrafish	Aquaporin-4 Isoform 1 [Danio Rerio]
NP_956204	Zebrafish	Aquaporin-7[Danio Rerio]
AAW64464.1	Zebrafish	Aquaporin 8 [Danio Rerio]
NP_001028268.1	Zebrafish	Aquaporin-9A [Danio Rerio]
AAH75911.1	Zebrafish	Aquaporin 10 [Danio Rerio]
NP_001314822.1	Zebrafish	Aquaporin-11 [Danio Rerio]
NP_001039327.1	Zebrafish	Aquaporin 12 [Danio Rerio]

Supplementary Table S2. Quality control and mapping statistics associated with individual sequencing libraries from the different blue mussel (*M. edulis*) treatments.

Treatment	Sample code	Raw reads[1]	Clean reads[2]	% Q20[3]	% GC[4]	% reads mapped[5]
	A1	23274003	22917823	98.18	34.56	81.28
	A2	24153587	23730425	97.24	34.45	82.23
	A3	31275830	30404027	97.53	34.53	81.69
23‰ 5°C	A4	33102473	32269107	97.44	34.56	82.61
(control)	A5	23397201	23231153	97.40	34.59	80.67
	B1	22493423	22138685	97.37	34.33	80.76
	B2	28756196	27971989	97.38	34.35	80.97
	B3	25309340	25000434	97.50	34.23	81.81
	B4	32698528	32236872	97.48	33.96	78.37
23‰ 30°C	B5	30632235	30284429	97.39	34.56	79.57
	C1	25897101	25453251	97.53	34.79	85.33
	C2	24378855	24068087	97.25	34.74	82.07
	C3	24025104	23481792	98.26	34.50	79.38
	C4	21500290	21108950	98.31	34.66	83.36
23‰ 33°C	C5	23003586	22666638	97.26	34.01	82.63
	D1	30225306	29742611	97.46	34.05	81.64
	D2	24215632	23868710	98.19	34.82	81.66
	D3	20009423	19639696	97.19	33.61	81.30
	D4	24677531	24166991	97.34	34.38	82.44
15‰ 5°C	D5	23255190	22885344	97.52	34.55	78.61
	E1	24594244	24306333	97.46	33.94	81.11
	E2	31904273	31494942	97.46	35.24	84.45
	E3	29302837	28828826	97.48	34.28	79.42
	E4	28276293	27761226	97.32	35.02	84.31
15‰ 30°C	E5	27448562	26909446	97.42	34.08	78.14
	F1	25408759	25037037	97.16	34.38	82.40
	F2	27247428	26668077	97.48	35.57	82.39
	F3	26886181	26378084	96.83	34.65	78.78
	F4	21893430	21255634	97.07	34.95	81.00
15‰ 33°C	F5	31325639	30819053	97.52	34.78	80.66
	G1	25752439	25285550	98.15	34.90	80.17
	G2	31105025	30784218	98.31	35.44	81.52
	G3	22102779	21752714	98.23	35.01	77.76
	G4	19136478	18925398	98.26	35.68	77.19
5‰ 5°C	G5	24805418	24470503	98.28	35.24	79.07

Average sequencing base error rate for all libraries was 0.03%

[1]Total number of raw reads
[2]Number of reads after filtering
[3]Percentage of bases with correct base recognitions rates are > 99% in total bases
[4]Percentages of G and C in total bases
[5] Percentage of library reads mapping back to the main transcriptome

Supplementary Table S3. Gene Ontology term (GO) enrichment results for upregulated gene expression in blue mussels (*M. edulis*). Abbreviations: BP: Biological process; CC: Cellular Component; MF: Molecular Function.

Category	ID	Description	Adjusted p-value
23‰ 30°C vs. 23‰ 5°C			
BP	GO:0000902	cell morphogenesis	3.62E-07
23‰ 33°C vs. 23‰ 5°C			
BP	GO:0000902	cell morphogenesis	1.64E-08
MF	GO:0003700	DNA-binding transcription factor activity	5.79E-07
BP	GO:0009058	biosynthetic process	4.23E-05
BP	GO:0034641	cellular nitrogen compound metabolic process	3.20E-03
BP	GO:0048856	anatomical structure development	3.20E-03
CC	GO:0005634	nucleus	3.26E-03
MF	GO:0019899	enzyme binding	3.41E-02
MF	GO:0051082	unfolded protein binding	3.43E-02
CC	GO:0005615	extracellular space	4.11E-02
15‰ 5°C vs. 23‰ 5°C			
BP	GO:0007155	cell adhesion	3.71E-02
CC	GO:0005576	extracellular region	3.71E-02
5‰ 5°C vs. 23‰ 5°C			
MF	GO:0003700	DNA-binding transcription factor activity	5.03E-03
BP	GO:0007155	cell adhesion	1.38E-02
CC	GO:0005856	cytoskeleton	3.30E-02
MF	GO:0003924	GTPase activity	3.55E-02
CC	GO:0005811	lipid droplet	3.55E-02
15‰ 33°C vs. 15‰ 30°C			
MF	GO:0003700	DNA-binding transcription factor activity	6.01E-03
BP	GO:0006457	protein folding	6.01E-03
BP	GO:0000902	cell morphogenesis	9.48E-03
BP	GO:0034641	cellular nitrogen compound metabolic process	1.61E-02
CC	GO:0005615	extracellular space	1.64E-02
BP	GO:0048856	anatomical structure development	2.74E-02
CC	GO:0005576	extracellular region	4.15E-02
15‰ 30°C vs. 15‰ 5°C			
BP	GO:0000902	cell morphogenesis	1.56E-09
BP	GO:0048856	anatomical structure development	4.41E-05
BP	GO:0006457	protein folding	2.71E-03
MF	GO:0051082	unfolded protein binding	2.71E-03
MF	GO:0043167	ion binding	4.03E-02

15‰ 33°C vs. 15‰ 5°C			
BP	GO:0000902	cell morphogenesis	1.46E-10
MF	GO:0051082	unfolded protein binding	5.50E-09
MF	GO:0003700	DNA-binding transcription factor activity	4.24E-08
BP	GO:0048856	anatomical structure development	1.55E-05
BP	GO:0006457	protein folding	3.16E-05
CC	GO:0005615	extracellular space	1.90E-04
BP	GO:0034641	cellular nitrogen compound metabolic process	7.32E-04
BP	GO:0009058	biosynthetic process	9.61E-04
BP	GO:0006950	response to stress	9.67E-04
BP	GO:0000278	mitotic cell cycle	1.01E-03
BP	GO:0007049	cell cycle	2.22E-03
CC	GO:0005886	plasma membrane	8.54E-03
CC	GO:0005783	endoplasmic reticulum	1.45E-02
BP	GO:0000003	reproduction	1.58E-02
MF	GO:0019899	enzyme binding	1.84E-02
CC	GO:0005634	nucleus	2.27E-02
BP	GO:0140014	mitotic nuclear division	2.51E-02
BP	GO:0042592	homeostatic process	3.28E-02
MF	GO:0016779	nucleotidyltransferase activity	3.72E-02
5‰ 5°C vs. 15‰ 5°C			
MF	GO:0003700	DNA-binding transcription factor activity	1.27E-03
BP	GO:0002376	immune system process	1.27E-03
BP	GO:0008283	cell proliferation	8.77E-03
BP	GO:0000003	reproduction	1.05E-02
BP	GO:0007049	cell cycle	1.05E-02
No enrichment			
23‰ 33°C vs. 23‰ 30°C			
15‰ 30°C vs. 23‰ 30°C			
15‰ 33°C vs. 23‰ 33°C			

Supplementary Table S4. Shared Gene Ontology term (GO) enrichment categories for upregulated gene expression between different blue mussels (*M. edulis*) treatment condition comparisons, highlighting the number of shared genes within shared GO terms for: (i) low salinity exposure, (ii) heat stress, (iii) heat stress under low salinity exposure, (iv) 30°C heat stress under low salinity exposure (v) 33°C heat stress under low salinity exposure

(i): Low salinity exposure

**Enriched GO category**	**GO ID**	**Description**	**No of genes**				
			Low salinity vs. control	Very low salinity vs. control	Very low salinity vs. Low salinity	Total	Shared
BP	GO:0007155	cell adhesion	14	46	N/A	60	9
MF	GO:0003700	DNA-binding transcription factor activity	N/A	62	41	103	24

(ii): Heat stress

**Enriched GO category**	**GO ID**	**Description**	**No of genes**				
			30°C vs. control	33°C vs. control	33°C vs. 30°C	Total	Shared
BP	GO:0000902	cell morphogenesis	15	17	N/A	32	13

(iii): Heat stress (30°C and 33°C) under low salinity exposure

**Enriched GO category**	**GO ID**	**Description**	**No of genes**				
			Low salinity+30°C vs. low salinity	Low salinity+33°C vs. low salinity	Low salinity+33°C vs. low salinity+30°C	Total	Shared
BP	GO:0000902	cell morphogenesis	15	23	10	48	10
BP	GO:0048856	anatomical structure development	22	39	23	84	17
BP	GO:0006457	protein folding	11	22	15	48	5
MF	GO:0051082	unfolded protein binding	9	23	15	47	6
BP	GO:0034641	cellular nitrogen compound metabolic process	N/A	221	158	379	81
MF	GO:0003700	DNA-binding transcription factor activity	N/A	42	26	68	14
CC	GO:0005615	extracellular space	N/A	20	13	33	9

(iv): 30°C heat stress under low salinity exposure

**Enriched GO category**	**GO ID**	**Description**	**No of genes**			
			Low salinity+30°C vs. low salinity	30*C vs. control	Total	Shared
BP	GO:0000902	cell morphogenesis	15	15	30	11

(v): 33°C heat stress under low salinity exposure

**Enriched GO category**	**GO ID**	**Description**	**No of genes**			
			Low salinity+33°C vs. low salinity	33°C vs. control	Total	Shared
BP	GO:0000902	cell morphogenesis	23	17	40	15
MF	GO:0051082	unfolded protein binding	23	9	32	5
MF	GO:0003700	DNA-binding transcription factor activity	42	31	73	19
BP	GO:0048856	anatomical structure development	39	24	63	19
CC	GO:0005615	extracellular space	20	11	31	7
BP	GO:0034641	cellular nitrogen compound metabolic process	221	147	368	57
BP	GO:0009058	biosynthetic process	181	131	312	50
MF	GO:0019899	enzyme binding	23	17	40	9
CC	GO:0005634	nucleus	98	73	171	26

Supplementary Figure S1. Primer sequences used in Nielsen et al. (2021) aligned with mapped transcripts from the blue mussel (*M. edulis*) transcriptome in this study. Shaded base pairs highlight agreements, a) *HSP70* primer alignment showing 100% identical sites to the best matching transcript, b) *HSP90* primer alignment showing 95% identical sites to the best matching transcripts, c) Heatmap showing relative fold change expression of identified transcripts using primer sequences. Cluster 53306.116803 (representing *HSP90*), and clusters 53306.127741 and 53306.129262 (representing *HSP70*) show similar expression patterns to the results seen in Nielsen et al. (2021). Red = High expression; Blue = Low expression.

a)

```
HSP 70 primer reverse                ----cctgcaatgaaaccagcatcc-----   21
Cluster-53306.127741 (reversed)      aagtcctgcaatgaaaccagcatcctttgtg  31
Cluster-53306.129262                 aagtcctgcaatgaaaccagcatcctttgtg  31
```

b)

```
HSP 90 primer                        ------tcagcaacaaggtaagcgga-------  20
Cluster-53306.116781 extraction      actttatcagaaacaaggtaagcggagtagaaa  33
Cluster-53306.117137 extraction      actttatcagaaacaaggtaagcggagtagaaa  33
Cluster-53306.116803 extraction      actttatcagaaacaaggtaagcggagtagaaa  33
Cluster-53306.116689 extraction      actttatcagaaacaaggtaagcggagtagaaa  33
Cluster-53306.116738 extraction      actttatcagaaacaaggtaagcggagtagaaa  33
```

c)

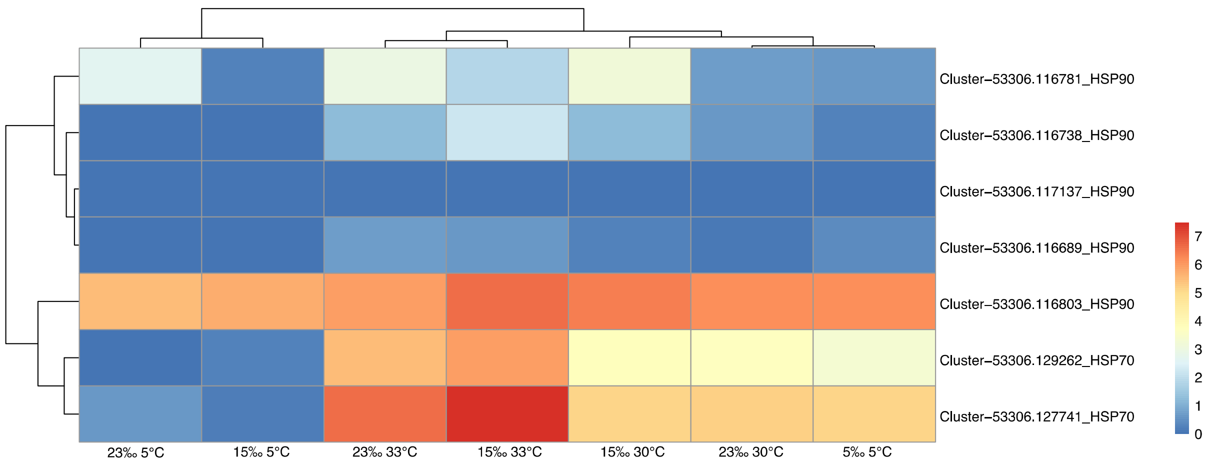

Supplement: Supplementary file 1 [file genes-13-00155-s001.zip › genes-1538545-supplementary/genes-1538545-supplementary.pdf]
